# A New Methodological Approach on the Characterization of Optimal Charging Rates at the Hydrogen Plasma Smelting Reduction Process Part 2: Results

**DOI:** 10.3390/ma15124065

**Published:** 2022-06-08

**Authors:** Daniel Ernst, Michael Andreas Zarl, Julian Cejka, Johannes Schenk

**Affiliations:** 1 Department of Metallurgy, Chair of Ferrous Metallurgy, Montanuniversitaet Leoben, Franz-Josef Str. 18, 8700 Leoben, Austria; julian.cejka@unileoben.ac.at (J.C.); johannes.schenk@unileoben.ac.at (J.S.); 2 K1-MET GmbH, Stahlstraße 14, 4020 Linz, Austria; michael.zarl@k1-met.com

**Keywords:** HPSR, kinetic, smelting reduction, iron ore, hydrogen reduction, plasma reduction

## Abstract

To meet the target for anthropogenic greenhouse gas (GHG) reduction, the European steel industry is obliged to reduce its emissions. A possible pathway to reach this requirement is through developments of new technologies for a GHG-free steel production. One of these processes is the hydrogen plasma smelting reduction (HPSR) developed since 1992 at the Chair of Ferrous Metallurgy at the Montanuniversitaet Leoben in Austria. Based on the already available publication of the methodology in this work, potential process parameters were investigated that influence the reduction kinetics during continuous charging to improve the process further. Preliminary tests with different charging rates and plasma gas compositions were carried out to investigate the impacts on the individual steps of the reduction process. In the main experiments, the obtained parameters were used to determine the effect of the pre-reduction degree on the kinetics and the hydrogen conversion. Finally, the preliminary and main trials were statistically evaluated using the program MODDE^®^ 13 Pro to identify the significant influences on reduction time, oxygen removal rate, and hydrogen conversion. High hydrogen utilization degrees could be achieved with high iron ore feeding rates and low hydrogen concentrations in the plasma gas composition. The subsequent low reduction degree and thus a high proportion of oxide melt leads to a high oxygen removal rate in the post-reduction phase and, consequently, short process times. Calculations of the reduction constant showed an average value of 1.13 × 10^−5^ kg oxygen/m^2^ s Pa, which is seven times higher than the value given in literature.

## 1. Introduction

The European Commission has committed to reduce greenhouse gas emissions by 80% in 2050 compared with 1990. One major emitter of CO_2_ is the steel industry, which is responsible for 7–9% of direct emissions from the global use of fossil fuels. In 2019, the EU28 produced 158.8 million tonnes of crude steel, of which 59.1% (93.9 million tonnes) was made via the integrated route (blast furnace–basic oxygen furnace) with specific estimated emissions of 1.85 t CO_2_/t steel and 40.9% (64.9 million tonnes) via the electric arc (EAF) route. Due to these emission levels, the iron and steel industry must develop new low-carbon technologies to meet the required greenhouse gas targets [1,2,3,4]. To further secure the steel production in Europe, the companies are focusing on two technological routes to reduce the CO_2_ emissions in the steel sector: Smart Carbon Usage (SCU) and Carbon Direct Avoidance (CDA). SCU includes process integration (PI) to modify existing fossil fuel-based processes and carbon capture and utilization (CCU) so that CO_2_ and waste gases can be used as raw materials for base chemicals manufacture. Carbon direct avoidance is dedicated to developing new processes and technologies to produce steel from iron ore without the direct release of carbon emissions. On the one hand, this includes electricity-based metallurgy with renewable energy and, on the other hand, hydrogen as the main reducing agent instead of carbon [1,5]. One of the possible key technologies for direct carbon avoidance is the Hydrogen Plasma Smelting Reduction (HPSR) process. With this method, the production of steel in one process step from pellet feed ores is achievable due to the high reduction potential of ionized and excited hydrogen. During the process, water vapor is emitted as a by-product, in contrast to coal-based processes where GHG’s are produced. The fundamentals of the HPSR process started in 1992 at the Montanuniversitaet Leoben and the further development of the process continues to this day [6,7,8,9,10,11]. An important aspect is the reduction kinetic, which significantly impacts the ecological and economic efficiency of the process. Various parameters such as the charging rate, the degree of pre-reduction, and the hydrogen content in the plasma gas strongly influence the reduction kinetics and the hydrogen utilization degree. To ensure economic efficiency, the exact influence of these individual factors must be determined to reduce process and plant costs. The aim of the study was to identify process parameters that influence the kinetics of the hydrogen plasma smelting reduction process to reduce the reduction times to a minimum while at the same time achieving high hydrogen utilization.

## 2. Equipment and Methods

The system components, the procedure description, and the evaluation method have already been explained in detail in other publications [12,13] and are only briefly mentioned here.

### 2.1. HPSR Laboratory Equipment

**Figure 1:**
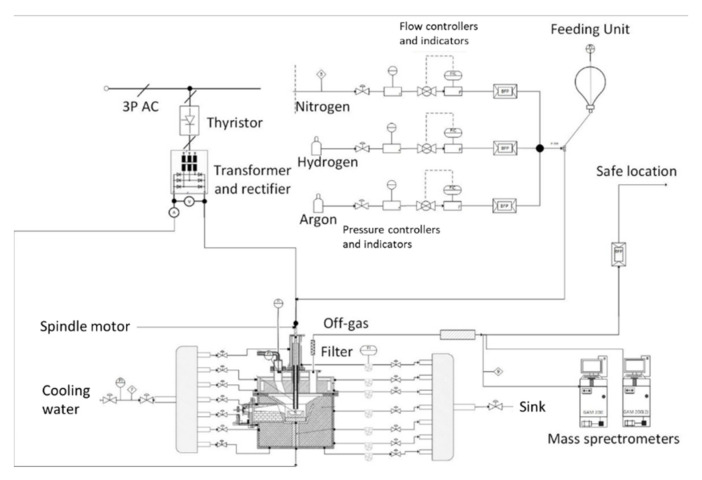
Flowsheet of the HPSR laboratory plant at the Montanuniversitaet in Leoben [12].

The experiments were conducted at the HPSR laboratory facilities at the Montanuniverstaet Leoben, Austria. The flowchart is shown in Figure 1. The reactor consisted of an upper and lower part lined with refractory material. The mode of the arc was DC transferred with an average power input of 5 kVA. The arc was ignited between a hollow graphite electrode (HGE) (with 10 to 26 mm outer diameter) and an ignition pin in a steel crucible. Through the HGE, the process gas (Ar, H_2_ and N_2_) and the ultra-fine ore were introduced into the reaction zone. The waste gas was cleaned by a hot gas filter from the Swedish–Swiss multinational corporation ABB^®^ Ltd. (Vasteros, Sweden) and was subsequently dehumidified to obtain a dry and dust-free gas stream to the mass spectrometer (Type GAM 200) from Pfeiffer Vacuum Technologies AG (Aßlar, Germany) to analyze the exhaust gas composition. To visually examine the process, a camera system AXIS-Q1775 from Axis Communications AB (Lund, Sweden) was used. 

### 2.2. Procedure Description 

**Figure 2:**
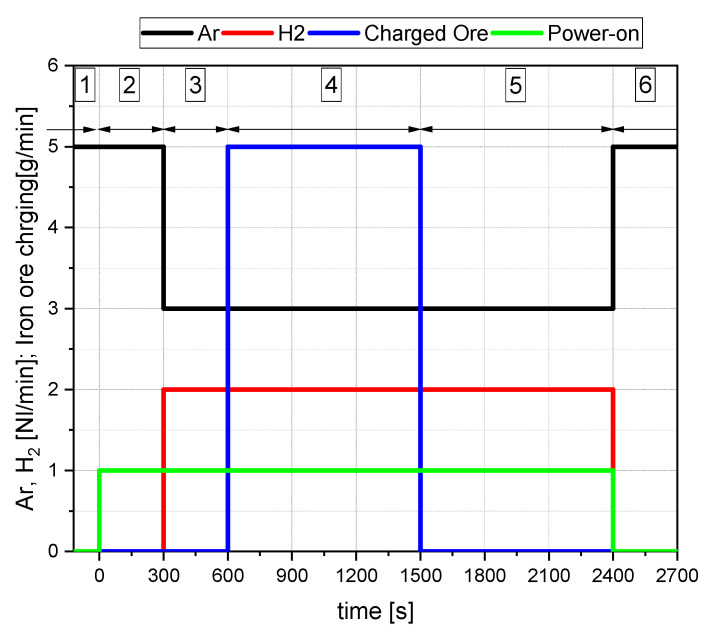
Example of an experimental procedure [14].

The procedure of the experiments can be described in six steps/phases (Figure 2):

1. Pure argon is injected into the system to create a completely inert atmosphere. In this phase, no electrical energy was supplied.
2. In the pre-melting step, the arc was lit between the HGE and the ignition pin in the steel crucible under pure argon to meltdown and reduce the batch-wise iron ore due to thermal decomposition.
3. With the start of the pre-reduction phase, the plasma gas composition was changed according to the experimental plan. In the preliminary tests, this phase was performed for 5 min. The time depends on the desired degree of pre-reduction for the main trials and can vary in the range of 1–5 min.
4. After the pre-reduction of the iron ore, the continuous charging of oxidic material through the HGE was carried out. For this purpose, the plasma gas composition and the charging rate were adjusted according to the trial program in Section 3.
5. The post-reduction phase aims to reduce the remaining oxide melt to metallic iron. This phase ended when the exhaust gas composition at the mass spectrometer showed no further increase in hydrogen.
6. The reactor was purged with argon to remove the remaining hydrogen as the last step.

### 2.3. Evaluation Methods

The mass spectrometer shows the volume percent of H_2_, Ar, N_2_, CO, CO_2_, and O_2_ of the detected exhaust gas. Nitrogen and oxygen were measured as well to ensure that the atmosphere is fully inert at step one. The conversion in absolute volume values was carried out via the argon ratio of its volume and its gas concentration. Therefore, the Ar volume flow, which was set with a mass flow controller, must first be multiplied with the time of the measurement interval of the mass spectrometer. For the equations of the produced steam, the mass of oxygen which is bound via CO, CO_2_, and H_2_O, and the degrees of conversion and reduction we refer to Part 1 of this paper [12], which has already been published.

## 3. Experimental Program

A total of three experiment series were carried out (Table 1 and Table 2). In the preliminary tests, the oxygen supply rate was varied via the charging speed (Ex. 1–X) and the oxygen removal rate via the hydrogen content was changed in the plasma gas (Ex. 2–X). In the preliminary tests, apart from the pre-melting period with pure argon, a constant hydrogen concentration was present throughout the entire process. Based on these trials, the progression of the pre-reduction and the equilibrium of the oxygen supply and removal during the charging phase were analyzed. On the control display of the charging system, the dosing speed range can be set between 0–999. Due to the specific densities of solid species, the power dosing speed depends on the powder properties. Based on experience, speed ranges for the used Carajas iron ore were suitable between 15 and 65.

Through this evaluation, the time and process parameters for the aimed pre-reduction degree were calculated to obtain a constant reduction degree during charging in the main experiments. The hydrogen content was partially reduced during the charging phase in the main trials. From these studies, the influence of the pre-reduction degree on the process time and hydrogen utilization will be determined.

Below, the composition and grain size distribution of the used Carajas iron ore, Table 3 and Table 4, the chemical composition of the steel crucible and ignition pin, Table 5, as well as the purity of the process gases, Table 6, are shown.

All other parameters were kept the same for all experiments. The pre-melting period always lasted 5 min and the post-reduction until no significant change could be detected in the off-gas analysis. From 100 g iron ore, 25 g were charged batch-wise in the crucible and 75 g were continuously charged. The gas flow was constant at 5 Nl/min and argon as well as its mixture with hydrogen was used as the carrier gas.

## 4. Results and Discussion 

### 4.1. Preliminary Tests and Parameter Definition for the Main Tests

The aim of the preliminary tests is to determine the oxygen input via the charged ore and the oxygen output via the hydrogen reacted to H_2_O. The main focus here is the pre-reduction degree and the curve progression of the reduction degree during the charging period. For this purpose, the charging rate is varied in the first series of tests with a constant hydrogen concentration in the plasma gas. In the second series, a varying hydrogen content of the supplied gas was used with a constant ore feed.

**Figure 3:**
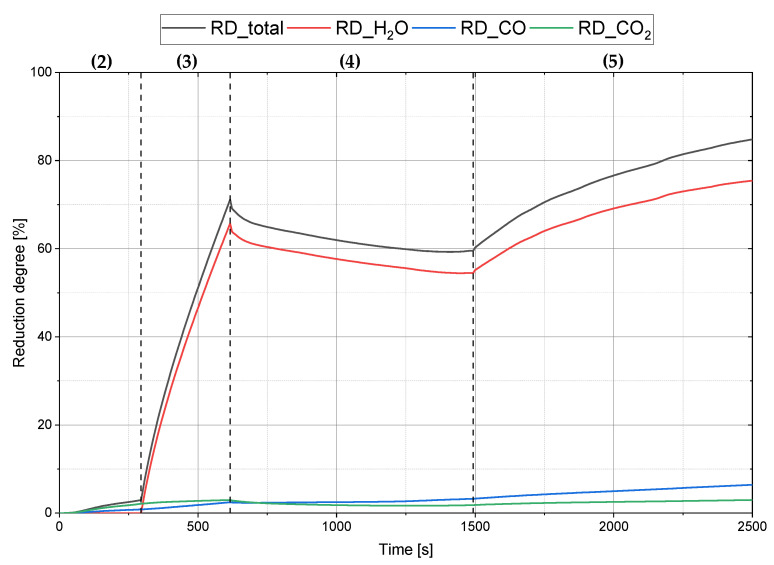
Reduction trend Ex. 1–3 with the associated procedure steps from chapter 2.2.

Figure 3 shows the individual progression of the reduction degrees from Ex. 1–3. The dashed line divides the diagram into individual experimental phases, from pre-melting the batch-wise iron ore to the end of the post-reduction phase. The gaseous carbon species were formed by the oxidation of the graphite cathode to carbon monoxide and further to carbon dioxide. After completing the pre-reduction, the proportion of the reduction degree by the formation of CO exceeded that of CO_2_. During charging, the total reduction degree declined due to the change in the ratio between supplied and removed oxygen. In the last stage, the post-reduction, the reduction degree rose with a more moderate slope compared with the pre-reduction phase due to the higher amount of iron ore in the reaction zone. Due to residues of iron ore in the charging system and small amounts of non-reduced material in the outer area of the crucible, a reduction degree of 100% was not achieved.

**Figure 4:**
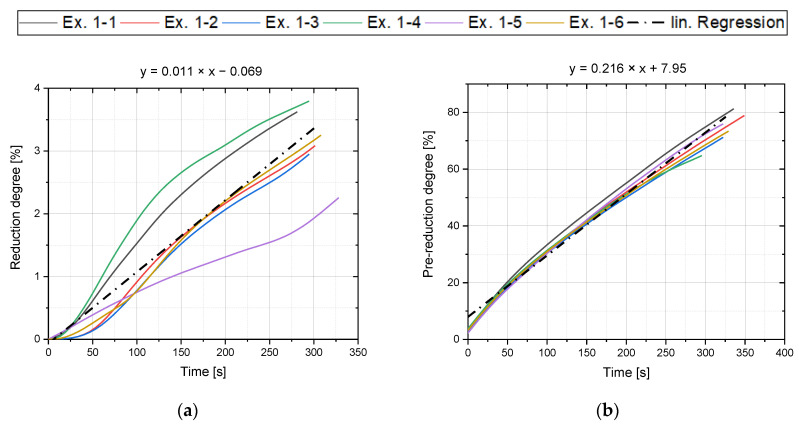
Pre-melting (**a**) and pre-reduction trend (**b**) Ex.1–1 to Ex. 1–6.

In the pre-melting phase, oxygen removal through thermal decomposition of hematite to magnetite occurred with pure argon and reduction degrees of approximately 3.5% were achieved. The comparison of the pre-melting procedure of the first preliminary series (Ex. 1–1 to Ex. 1–6) can be seen in Figure 4a. Another objective of the preliminary trials was to determine the necessary pre-reduction time to obtain specific degrees for the main trials. For this purpose, the progressions of the individual experiments were determined and a regression line was created, (Figure 4b), to subsequently calculate the required time.

**Figure 5:**
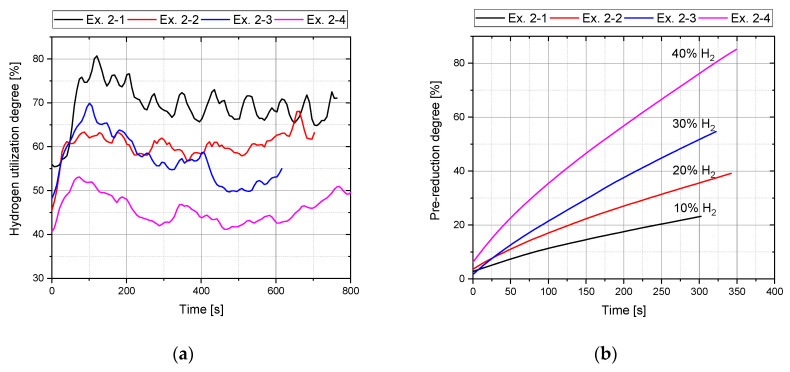
(**a**) Hydrogen utilization (Phase 4) and (**b**) Pre-reduction degree (Phase 3) Ex. 2–1 to Ex. 2–4.

The second preliminary series, with a change in the hydrogen content of the supplied gas at a constant ore feed rate, shows the influence on the hydrogen utilization degree during the charging phase, Figure 5a, as well as the impact on the oxygen removal rate during the pre-reduction phase, (Figure 5b). Lower hydrogen content in the plasma gas during the continuous iron ore feeding resulted in higher H_2_ utilization degrees. Several factors can explain the fluctuations in the graph of conversion. The design of the feed system does not result in a completely continuous ore feed into the reaction zone, but rather relatively small amounts of ore are fed in at short intervals. Additionally, the movement of the arc and thus the position of the molten bath affect the utilization degree as well. In case of an eccentric shift of the plasma arc, the iron ore charged to the center of the crucible cannot be melted and reduced. As a result, the degree of hydrogen conversion is negatively influenced. This leads to fluctuation of the graph. Based on Figure 5b, the variation of hydrogen in the process gas confirms that the kinetics in the initial reduction phase relied strongly on hydrogen transport. As the hydrogen content increased, more molecular, excited, and ionized hydrogen species were transported to the reaction zone and oxygen was removed more efficiently.

**Figure 6:**
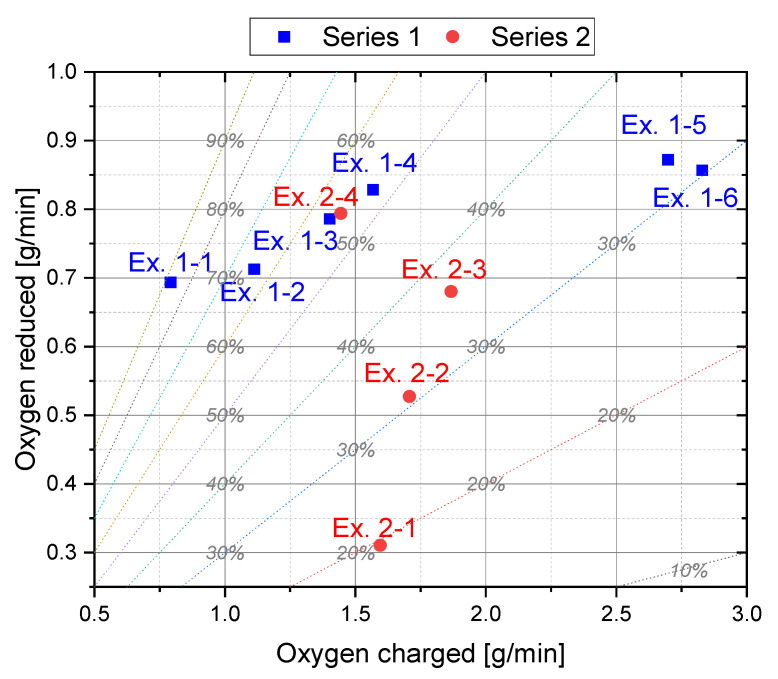
Reduced and charged oxygen during the charging phase (Phase 4).

In Figure 6, the reduced and charged oxygen during iron ore feeding is plotted for all preliminary trials. The oxygen removal rate includes the oxidation of hydrogen (H_2_O) and carbon species (CO and CO_2_). In addition, the dashed lines correspond to constant reduction degrees. Setting constant reduction degrees via the speed of the charging system is difficult due to the inconsistent input of iron ore at same feeding settings. This occurs due to the cohesive behavior of the material and bridging between the individual particles. Additionally, it is noticeable that the flow behavior of the iron ore was strongly influenced by the revolution speed of the discharge screw so that above a certain value an increased iron ore input could be detected, see difference in the charged oxygen at Ex. 1–4 and Ex. 1–5. This uneven supply of iron ore in the system again influenced the oxygen removal rate since it is dependent on the amount of oxygen introduced, as shown later. Therefore, setting the desired reduction degree during charging proved to be problematic in the subsequent main tests.

### 4.2. Main Experiments

**Figure 7:**
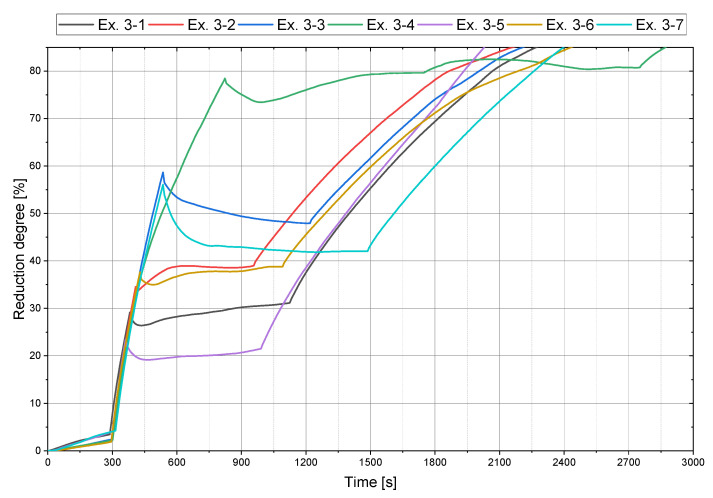
Reduction trend for the main trials.

The focus of the main tests, Figure 7, was to obtain a constant reduction degree during the charging phase to determine the influence on the post-reduction. The necessary pre-reduction time via the linear regression (Figure 4b) and the process parameters during feeding via the equilibrium of the charged and removed oxygen (Figure 6) were obtained to achieve constant values. 

**Figure 8:**
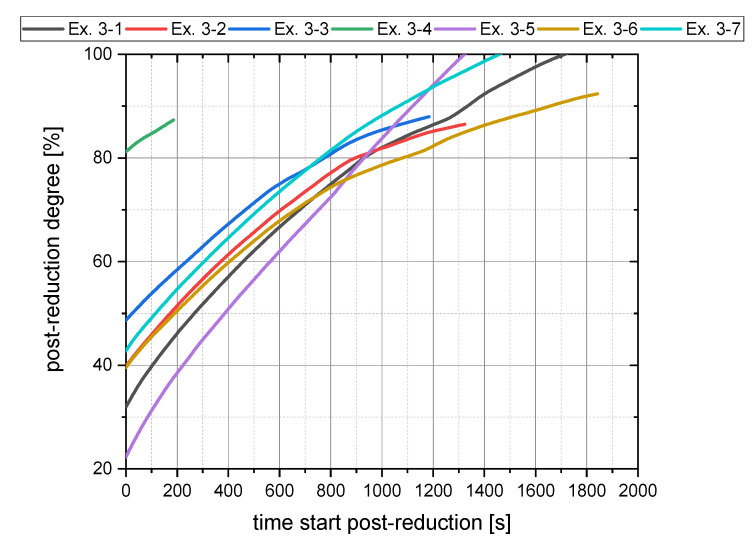
Post-reduction (Phase 5) trend for the main trials.

Considering the slope of the post-reduction for Ex. 3–1 to Ex. 3–7, it is noticeable that higher reduction degrees after iron ore charging led to a flatter curve during post-reduction, see Figure 8. Due to the higher proportion of oxide melt that can react with the hydrogen at the interface, there was a greater rate of oxygen removal.

At a reduction degree of about 80%, the determining step of the reduction changed, which is indicated by a change in the curve progression. From this moment, the kinetic did not rely on hydrogen transport to the reaction zone anymore but depended only on the oxygen transport in the melt to the reduction interface. The jet effect of the plasma arc, thermal flows within the molten bath, and the electromagnetic stirring resulted in the mixing of the metallic and oxide phases. Consequently, not enough dissolved oxygen was transported to the interface, which limited the reduction progress.

**Figure 9:**
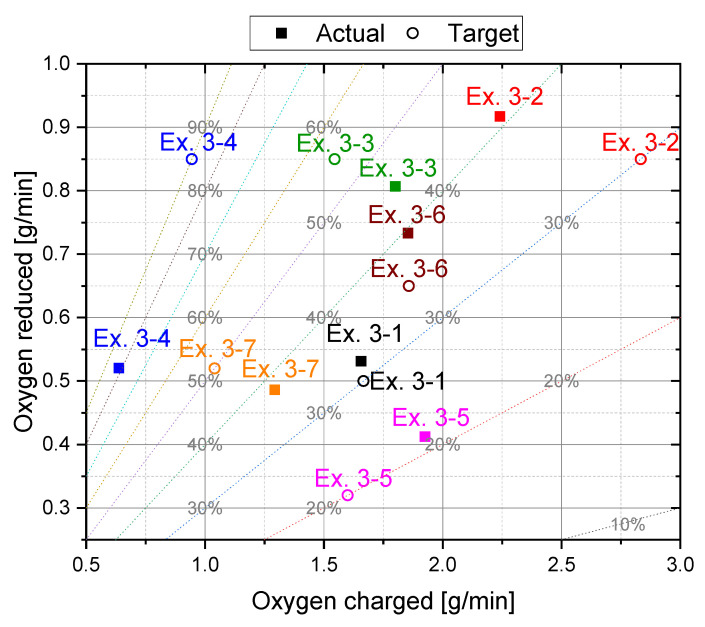
Reduced and charged oxygen during the charging phase for the main trials.

The previously mentioned scattering of the charging rate at constant feeding speed also led to deviations of the desired degree of reduction in this trial series. Figure 9 shows actual and target values for the reduced and charged oxygen during the charging phase for each experiment. The dashed lines show the constant reduction degrees again.

**Figure 10:**
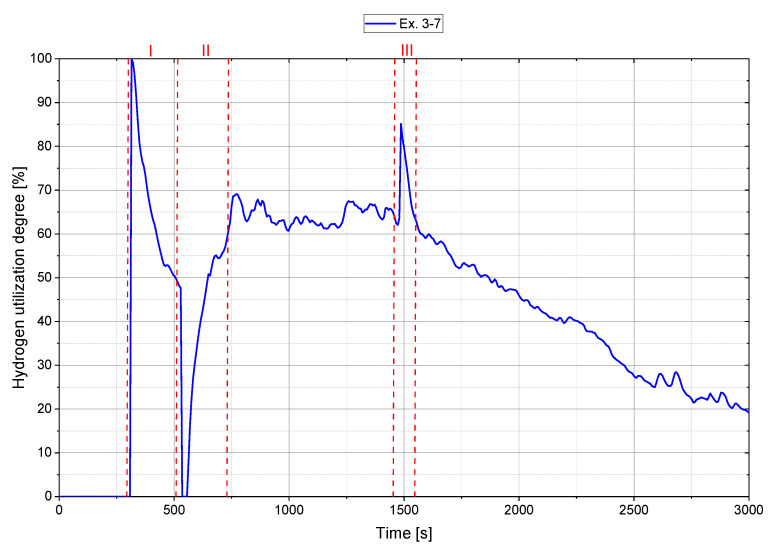
Trend of the hydrogen utilization degree for Ex. 3–7.

In Ex. 3–1 as well as in Ex. 3–5 to Ex. 3–7 the hydrogen content during iron ore feeding was changed and resulted in three peaks in the evaluation of the hydrogen utilization degree, which can be seen from Ex. 3–7, Figure 10. In peak I, when hydrogen was added to the reaction zone for pre-reduction, the mass spectrometer detected the H_2_ content with a delay. The reason for this is, on the one hand, the volume of the reactor and exhaust system, through the gas exchange between hydrogen and the therein contained argon. On the other hand, the spectrometer’s measuring interval recorded the exhaust gas composition every 6.7 s. This means that hydrogen was already available for the reduction but was not yet detected in the exhaust gas, which resulted in an apparent utilization rate of 100%. The reverse mechanism occurred when the hydrogen content was reduced for the charging phase, peak II. Higher H_2_ contents in the exhaust gas were detected by the spectrometer compared with the available content in the reaction zone. This resulted in conversion rates of 0% in the calculation. After a settling time, however, an equilibrium was reached between the introduced and detected hydrogen in the off-gas. At the last peak (III), the exact mechanism described at peak (I) occurred when the hydrogen content increased. To calculate the average conversion degree during charging, only the nearly constant range (between II and III) was used.

**Figure 11:**
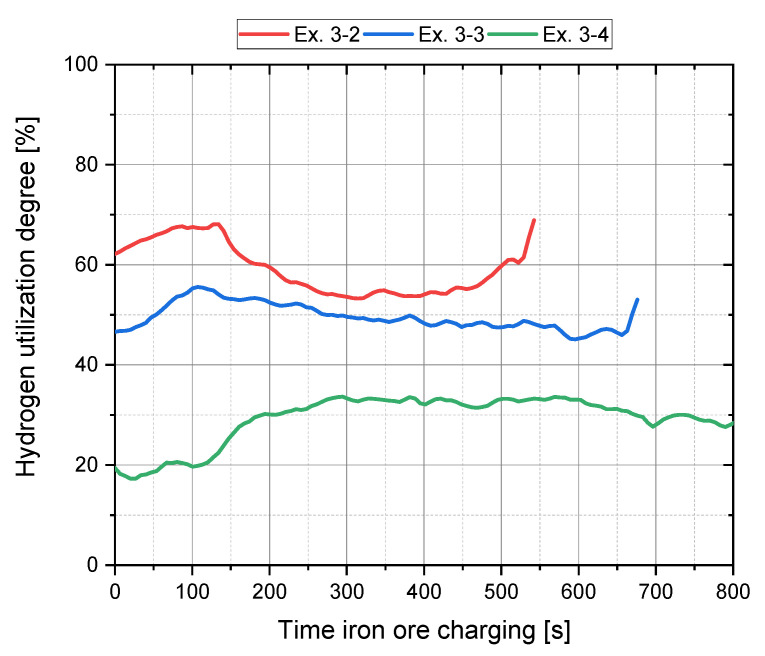
Hydrogen utilization during charging (Phase 4) for Ex. 3–2 to Ex. 3–4.

Further parameters which affect the hydrogen utilization degree are the charging rate and the degree of pre-reduction. Figure 11 illustrates the H_2_ utilization for Ex. 3–2 to Ex. 3–4, during iron ore feeding, with a constant hydrogen content from pre- to post-reduction and various charging rates as well as reduction degrees. It can be deduced that lower degrees of the reduction degree and higher iron ore charging rates lead to greater hydrogen usage.

**Figure 12:**
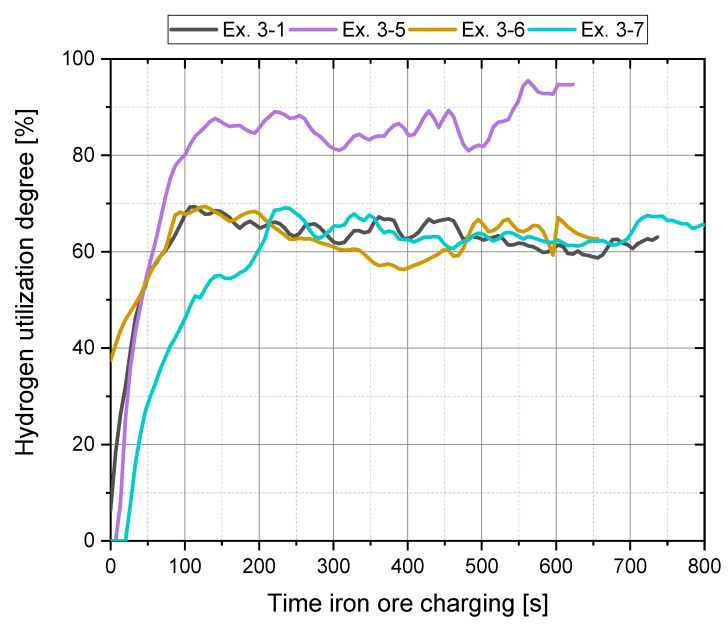
Hydrogen utilization during charging (Phase 4) for Ex. 3–1 and Ex. 3–5 to Ex. 3–7.

As already determined in the preliminary experiments, chapter 4.1, high H_2_ utilization degrees can be achieved if the proportion of hydrogen in the plasma gas is reduced during iron ore feeding, Figure 12.

### 4.3. Statistical Analysis

The preliminary and main experiments were also evaluated with the program MODDE^®^ 13 Pro from Sartorius AG to analyze the significant main effects on the hydrogen utilization degree and reduction time to achieve a reduction degree of 85%. For this purpose, the variable process parameters were first defined as factors and the output as responses, Table 7. Afterward, the general settings such as the individual factors type, range, and precision must be set. Outlier experiments responses, highlighted in gray, were excluded for the analysis as these deviate strongly. The time of reduction for a degree of 85% was excluded for Ex. 1–1 because of the high pre-reduction degree and low charging rate, and this limit value was already reached during the iron ore feeding. For this series of experiments MODDE additionally recommended excluding the oxygen reduction rate for this experiment. At trial Ex. 3–8, Carajas iron ore with an increased charging rate was added to obtain a larger oxygen input per time unit. 

#### 4.3.1. Hydrogen Utilization

**Figure 13:**
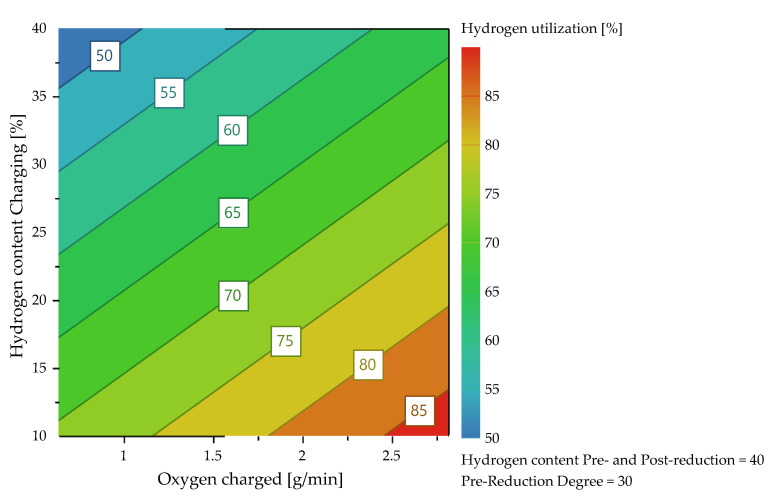
Average hydrogen utilization as a function of the H_2_ content and the charging rate.

**Figure 14:**
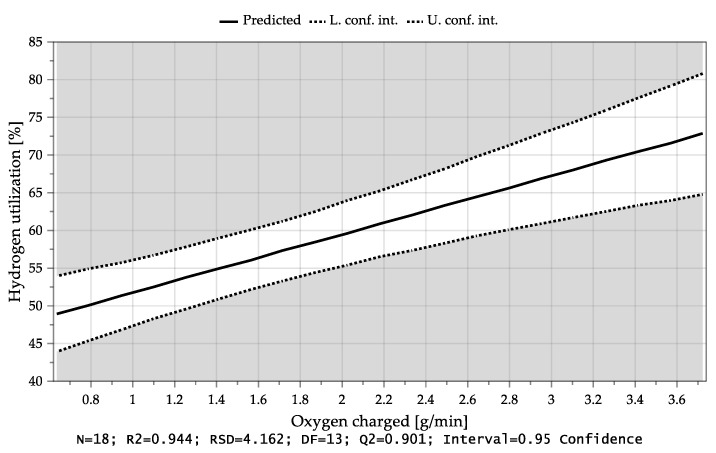
Average hydrogen utilization as a function of the charged oxygen rate.

**Figure 15:**
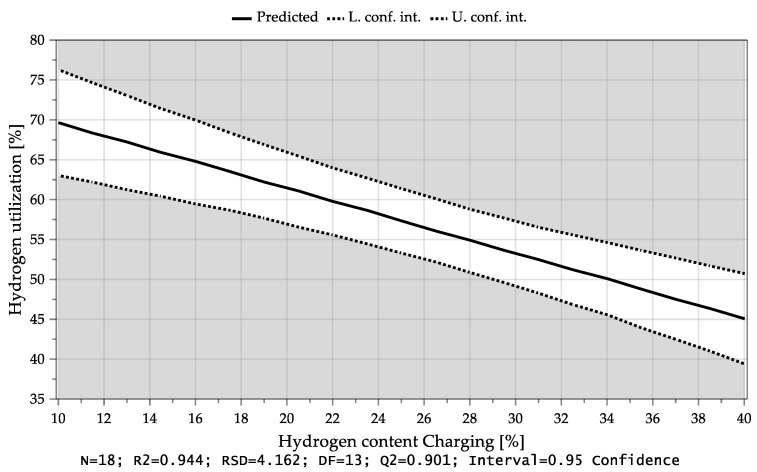
Average hydrogen utilization as a function of the H_2_ content during iron ore charging.

MODDE^®^ 13 Pro validates the shape of the response distribution and applies a transformation which was not necessary in this case. Subsequently, the significant coefficients of the individual responses were identified and all unimportant terms were removed. Table 8 explains the acronyms appearing in the diagrams. Figure 13 shows the dependency of the average hydrogen utilization degree during charging of both factors. In the lower right part of the diagram, the parameters that were kept constant are shown in percent. Here, it is evident that high conversion levels could be achieved with a low H_2_ content in the plasma gas during charging and a high feeding rate. It can be seen that the average hydrogen utilization degree during the iron ore charging depended mainly on the charging rate and thus on the oxygen input as well as the hydrogen content during iron ore feeding, Figure 14 and Figure 15. If more oxide material is charged into the reaction zone per time unit, a higher amount of hydrogen can react with the oxygen of the dissociated iron oxides at the reaction interface. As a result, the degree of utilization increases. Higher hydrogen content in the plasma gas leads to higher oxygen removal rates, which also results in larger amounts of water vapor at the phase boundary. After desorption, H_2_O is transported through the phase boundary layer and collides with the newly introduced hydrogen, which prevents the hydrogen from being adsorbed again and thus, it cannot take part in the reduction. 

**Figure 16:**
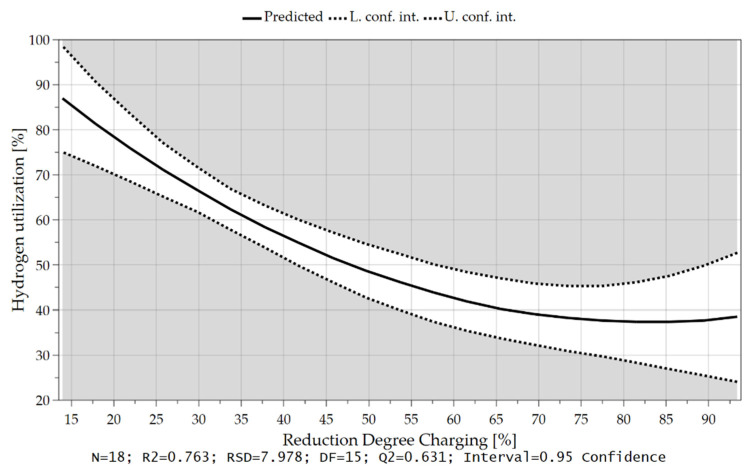
Hydrogen utilization as a function of the Reduction Degree during iron ore feeding.

The higher the iron ore feed rate (high oxygen supply) and the lower the hydrogen content in the plasma gas during charging (low oxygen reduction), the lower the equilibrium reduction degree. Low reduction degrees lead to higher hydrogen utilization, which is also shown in Figure 16.

**Figure 17:**
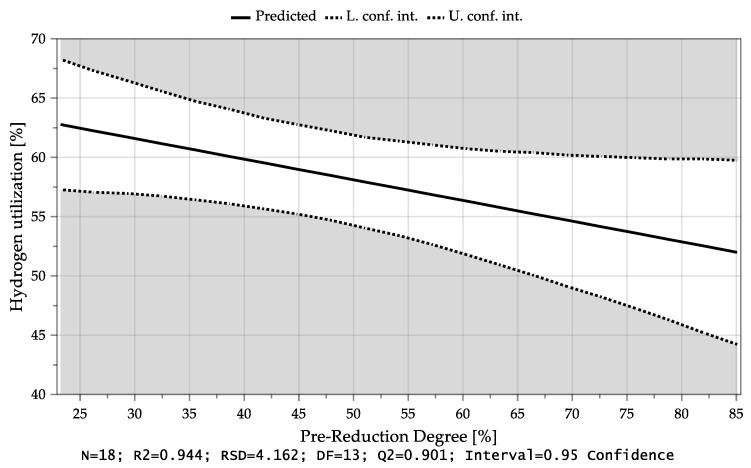
Average hydrogen utilization as a function of the Pre-reduction Degree.

As mentioned before, the pre-reduction degree also influenced the H_2_ utilization, Figure 17, but not as significant as the added hydrogen or oxygen. 

#### 4.3.2. Oxygen Removal Rate during Continuous Charging

**Figure 18:**
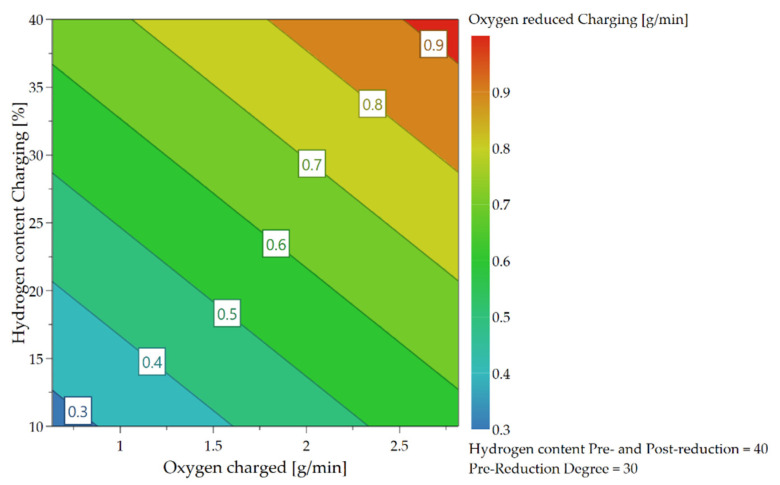
Dependence of oxygen removal rate on H_2_ content and oxygen supply during the charging phase.

**Figure 19:**
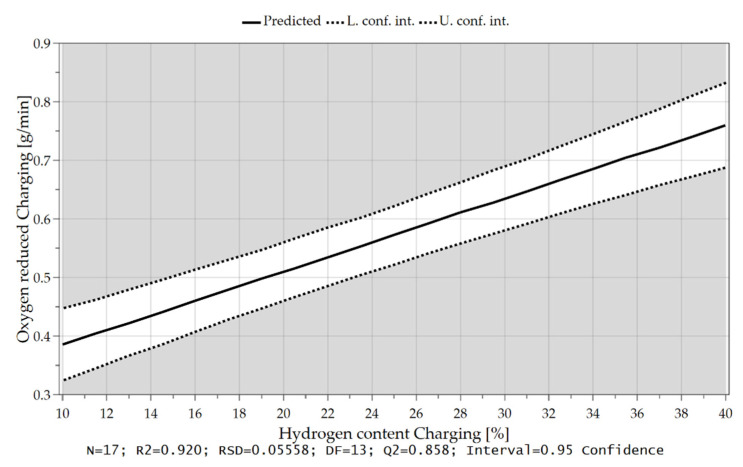
Oxygen removal rate as a function of the hydrogen content during charging.

**Figure 20:**
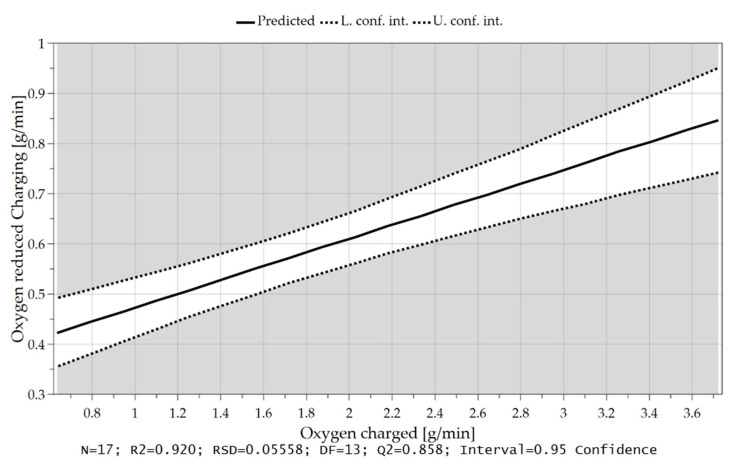
Oxygen removal rate as a function of the introduced oxygen via iron ore feeding.

As already briefly explained in chapter 4.1, the oxygen which can be removed per time unit depends not only on the hydrogen content in the feeding sequence but also on the charging rate and, therefore, on the introduced oxygen via iron ore. Due to the higher supply of oxygen at the reduction interface and the higher content of hydrogen, those are the rate-determining step of the kinetics, more oxygen can be bound to the hydrogen species and be removed. These influences and their combination are illustrated in the following Figure 18, Figure 19 and Figure 20.

#### 4.3.3. Oxygen Removal Rate during Post-Reduction

**Figure 21:**
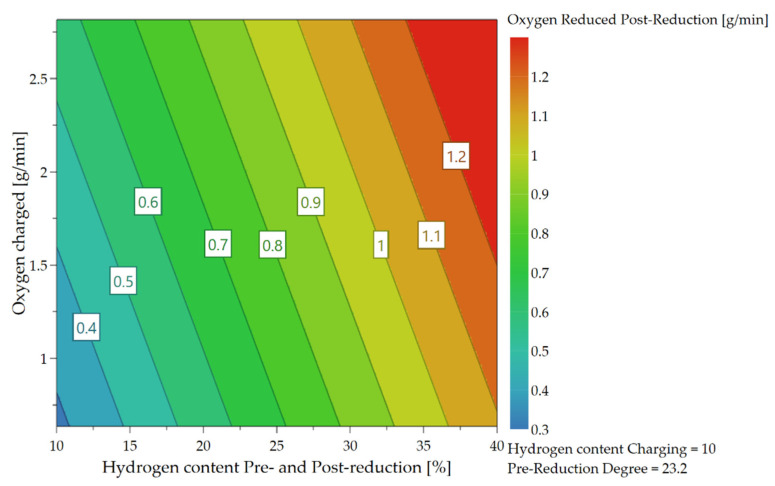
Dependence of the oxygen removal rate during the post-reduction process.

**Figure 22:**
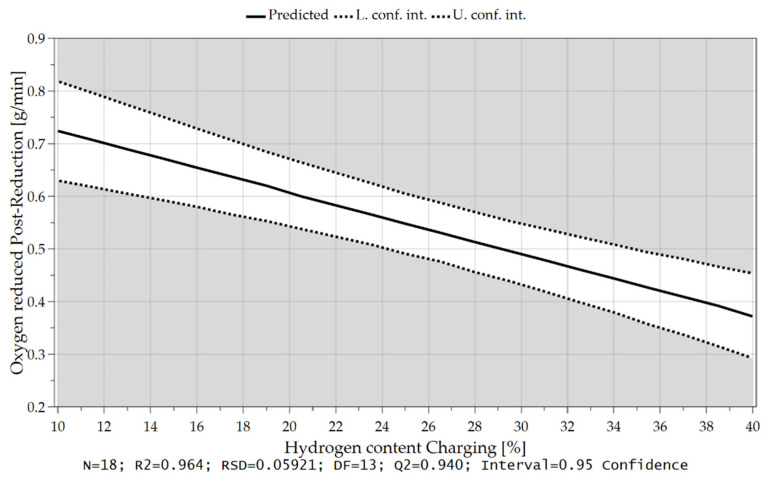
Oxygen removal rate in the post-reduction depending on the hydrogen content during iron ore feeding.

**Figure 23:**
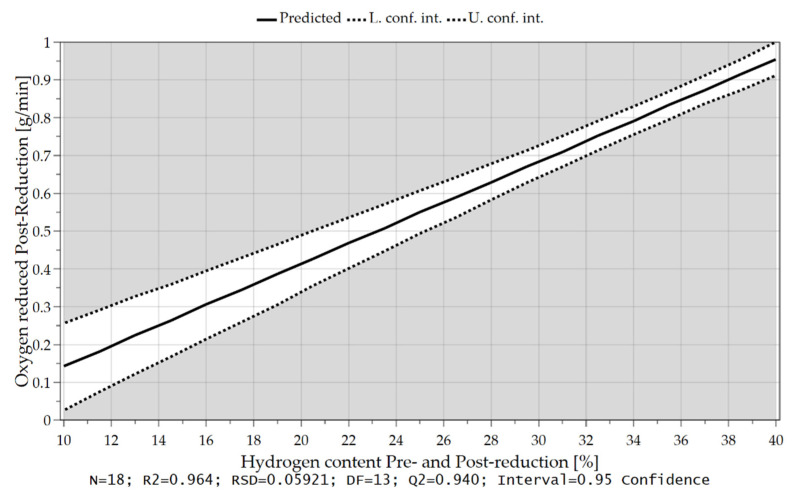
Oxygen removal rate in the post-reduction as a function of hydrogen content during post-reduction.

**Figure 24:**
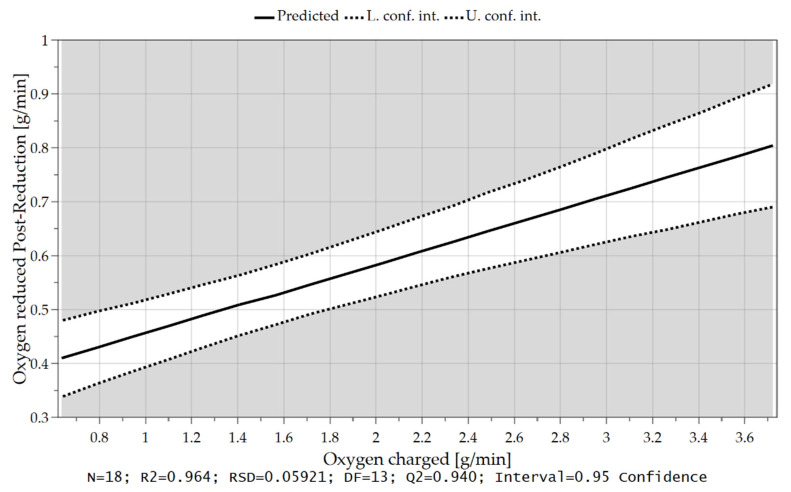
Oxygen removal rate in the post-reduction depending on the introduced oxygen through iron ore charging.

In Chapter 4.2 it was already mentioned that at the beginning of the post reduction, a steeper reduction progression was observed at lower reduction degrees after iron ore feeding. Statistical analysis also confirms this theory. After charging, the oxygen removal rate depends on the amount of hydrogen in the plasma gas and the proportion of oxidic melt. Assuming that the hydrogen content is reduced during continuous feeding and the iron ore is fed at a high rate, more oxygen is introduced into the reaction zone and a high proportion of oxide melt is present at the end of this phase. High oxygen removal rates are achieved if the fraction of H_2_ is increased again in the post-reduction, see Figure 21, Figure 22, Figure 23 and Figure 24.

### 4.4. Reaction Rate Constant for Hydrogen Reduction

The reduction rate *r* describes the oxygen removal rate for the reduction of liquid iron oxide melt with hydrogen related to the reaction area in the plasma focal hot spot [15].

(1) $$r=k_{a}\cdot \left(P_{H_{2}}-\frac{P_{H_{2}O}}{K_{H}^{\prime }}\right)$$

*r* Reaction rate (kg oxygen/m^2^ s)

$k_{a}$ Reaction rate constant Wüstite/H_2_ (kg oxygen/m^2^ s Pa)

$P_{H_{2}}$ Partial pressure of hydrogen (Pa)

$P_{H_{2}O}$ Partial pressure of water (Pa)

$K_{H}^{\prime }$ Partial pressure ratio ($P_{H_{2}O}$)/($P_{H_{2}}$) in equilibrium

In Part 1 of this publication, the method to measure the dimensions of the plasma focal spot and the related calculation for the determination of $k_{a}$ was explained. Rearranging of Equation (1) and introducing the focal spot area A results in

(2) $$k_{a}=\frac{\frac{r^{\prime }}{A}}{\left(P_{H_{2}}-\frac{P_{H_{2}O}}{K_{H}^{\prime }}\right)}$$

*r*’ Oxygen removal rate [kg oxygen/s]

*A* Area of the focal spot [m^2^]

Experiments Ex. 1–4, Ex. 1–6, and Ex. 3–3 were used for the determination of the reaction rate constant $k_{a}$ since an H_2_ content of 40% was used during charging and similar oxygen removal rates were achieved. For this purpose, five images per trial were analyzed and the areas of the focal spots were determined. Table 9 shows the focal spot areas as well as the measured and calculated parameters during iron ore feeding for these three experiments. 

For the determination of $K_{H}^{\prime }$ is assumed that the reduction of iron oxide with hydrogen occurs solely in the focal spot and the gas mixture leaving is in the equilibrium. Consequently, the measured GOD of the off-gas represents the equilibrium partial pressure ratio of ($P_{H_{2}O}$)/($P_{H_{2}}$) in the focal spot. With this information the temperature in the focal spot can be estimated using the Baur–Glaessner diagram.

**Figure 25:**
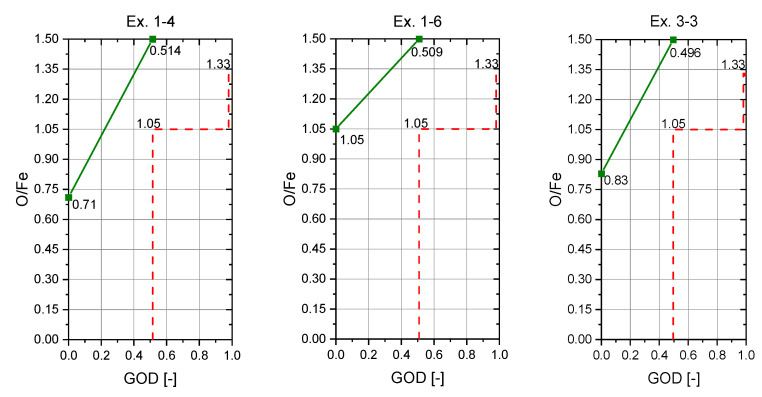
RIST diagrams for Ex. 1–4, Ex. 1–6, and Ex. 3–3.

The determination of the exact focal spot area for each experiment proved to be difficult. As shown in Table 9, these varied strongly, and deviations up to a factor of 3.3 (Ex. 3–3) were measured. All three trials had similar oxygen removal rates and the same hydrogen supply of 40% (2 L/min) in the plasma gas. Significant differences were evident in the oxygen supply rate and consequently also in the specific hydrogen supply per gram of oxygen charged via the iron ore. This difference was also reflected by the slope of the operation line (green) of the three experiments in the RIST diagrams, Figure 25. The steepness of the slope indicated the reducing agent demand. This resulted, too, in a varying equilibrium reduction degree during charging, which is indicated in the RIST diagram by the O/Fe ratio at the ordinate intercept. The red dashed line represent the version of the RIST diagram for the HPSR reactor. In this case, thermal decomposition is neglected and the hematite is reduced to metallic iron. Despite this difference in the amount of charged material per time unit, the gas utilization of the three experiments was quite identical and therefore also the GOD. As mentioned above, the GOD represents the equilibrium partial pressure ratio of (p_H2O_)/(p_H2_) in the focal spot, so they were not significantly different from each other. The calculated reaction rate constant for the three experiments was between 9.39 × 10^−6^ and 1.35 × 10^−5^ kg oxygen/m^2^ s Pa, which is mainly caused by the different determined focal spot sizes. It resulted in an average value of 1.13 × 10^−5^ kg oxygen/m^2^ s Pa. This value is by a factor of 7 higher than that of Nagasaka et al. [15]. The higher temperatures present in some cases, as well as the use of plasma, can be assumed as the reason for this.

**Figure 26:**
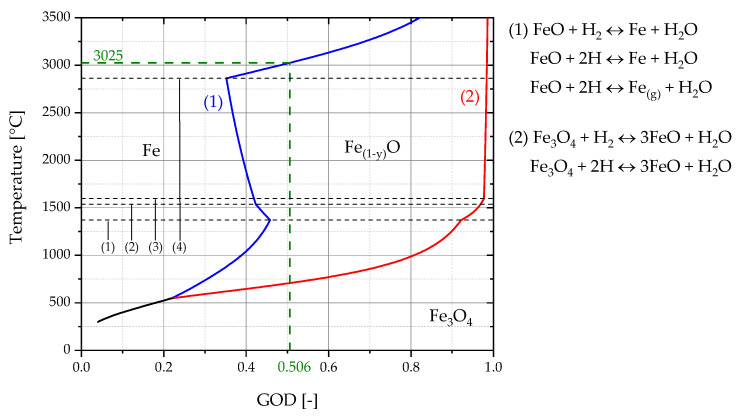
Baur–Glaessner diagram calculated with FactSage^TM^ 8.0. Database: FToxid, FactPS.

The obtained GOD’s were additionally transferred to a calculated Baur–Glaessner phase stability diagram to estimate the average temperature in the focal spot. The phase boundary lines between Fe-Fe_(1−y)_O and Fe_(1−y)_-Fe_3_O_4_ in Figure 26 were calculated using the FactSage^TM^ 8.0 Database: FToxid, FactPS Reaction Module. In order to improve readability, the averaged GOD of the three experiments, 0.506, is shown here. Using this value a plasma temperature of 3025 °C could be determined, as shown in Figure 26 (green dashed lines). For this estimation, it is assumed that the system is in equilibrium. The black dashed lines indicate the phase transitions of each species:

(1) 1371 °C  FeO(s) → FeO(l)
(2) 1538 °C  Fe(s) → Fe(l)
(3) 1597 °C  Fe_3_O_4_(s) → Fe_3_O_4_(l)
(4) 2862 °C  Fe(l) → Fe(g)

## 5. Conclusions and Outlook 

### 5.1. Conclusions

#### 5.1.1. Experimental Results

The pre-melting stage showed a reduction degree of approximately 3.5% due to thermal decomposition towards magnetite. Higher hydrogen utilization degrees were observed at reduced H_2_ contents in the plasma gas. However, longer reduction times also occurred due to the reduced oxygen removal. It was obtained that high reduction rates, above 80%, led to an unstable process. An explanation therefore is that accompanying oxides with high melting points (SiO_2_, Al_2_O_3_, CaO) enhance the liquidus temperature with decreasing FeO content. To obtain a specific reduction degree, the variation of the hydrogen content is preferable because of the fluctuating input of iron ore at constant charging rates. However, the oxygen removal rate is a function of the charging rate.

The main experiments revealed that the lower the reduction degree after the charging phase, the steeper the curve at the beginning of post-reduction. Due to this phenomenon, the total reduction time was not affected despite a lower reduction degree after charging. At a reduction degree of approximately 80%, the kinetic depended only on the oxygen transport of the dissociated iron oxides to the phase boundary. The amount of hydrogen was no longer a determining factor. The combination of low pre-reduction degrees and high charging rates led to higher conversion rates in the charging phase.

The statistical analysis software MODDE^®^ 13 Pro calculated the main effects on the H_2_ utilization and oxygen removal rate during the charging and post-reduction phase. This analysis found that the hydrogen utilization depended on the charging rate and H_2_ content during iron ore feeding. The degree of pre-reduction thereby had only a subordinate role. The oxygen removal rate during the continuous addition of iron ore depended not only on the H_2_ proportion but also on the supply rate of oxygen. In the post-reduction step, the hydrogen content in the plasma gas and the proportion of oxidic melt were decisive for the initial oxygen removal rate. These findings showed that low hydrogen contents during high iron ore feed rates led to high hydrogen utilization rates. The subsequent reduction should be carried out at the highest possible H_2_ content in the plasma gas to achieve short process times.

In determining the exact focal spot area for three experiments with similar H_2_ content of 40% during charging and similar oxygen removal rates, the calculated rate constant for hydrogen reduction ranged from 9.39 × 10^−6^ to 1.35 × 10^−5^ kg oxygen/m^2^ s Pa, yielding an average value of 1.13 × 10^−5^ kg oxygen/m^2^ s Pa.

#### 5.1.2. Comparison with the Results Reported in the Literature 

Due to the unique characteristic of the laboratory plant at the Montanuniversitaet Leoben as well as the combination of batch and continuous charged iron ore and the division of the reduction into different phases, a comprehensive comparison of the results is difficult. Nevertheless, influences of individual parameters on the reduction and utilization of the plasma gas can be compared. 

Nakamura et al. [16] determined that high utilization rates of the gas can be achieved at low hydrogen concentrations in the plasma gas. They showed that hydrogen utilization efficiencies as high as 70% can be achieved at particularly low H_2_ concentrations of 10% with a total gas flow of 10 Nl/min and a ore mass of 500 g. These results are in very good agreement with our investigations and, as in our case, lowering the hydrogen concentration over the entire process also had a negative effect on its duration and thus on its industrial usability.

Souza et al. [17] found that for a reduction of 9 g iron ore with a Ar-10%H_2_ plasma gas a 75% reduction was obtained within the first 15 min, while the last 25% also required the same time. Here, too, it was found that there is a change in the reduction kinetics and a decrease in the reduction rate at higher reduction levels. They also showed that the reduction rate was almost doubled from 1.08 g/min to 1.8 g/min by increasing the amount of iron ore from 9 g to 15 g. Our results also confirmed that higher oxygen removal rates could be achieved at higher proportions of oxidic melt for both the charging and post-reduction phases.

Seftejani [10] determined the decrease in hydrogen conversion degree throughout reduction. Using 100 g of iron ore and a gas mixture of 50% H_2_ and 50% Ar, he achieved a conversion rate at the beginning of the reduction of 44–58%, and at 50 g only less than 40%. He also confirmed that lower H_2_ utilization degrees were achievable with lower iron ore input and thus a lower proportion of oxide melt.

Kaminya et al. [18] showed the effect of a varying hydrogen concentration in an H_2_-Ar plasma on the reduction rate at constant a power input of 8.3 kW and a gas flow of 20 1/mm. They determined, like us, that the reduction rate increases with higher hydrogen content in the plasma gas. However, contrary to our results, the different H_2_ content did not influence the hydrogen utilization degree.

Nagasaka et al. [15] determined the constant rate for hydrogen reduction of 1.6 × 10^−6^ kg-oxygen/m^2^ s Pa at 1673 K. For argon–hydrogen mixtures (60%/40%) used in our experiments, an average value of 1.13 × 10^−5^ kg-oxygen/s Pa at 3025 °C was determined, which is seven times higher than the value given in the literature.

#### 5.1.3. Ecological Aspect

The experiments have shown that high feed rates of iron ore and low hydrogen concentrations lead to high H_2_ conversion rates during the feeding phase. The resulting low reduction degree in the post-reduction phase leads to a high oxygen removal rate and a fast conversion to metallic iron, so that short process times can be achieved. With this mode of operation, the hydrogen demand can be optimally utilized and does not have to be fed unused into the exhaust gas or recycled in large quantities. This saves costs due to the lower quantity of hydrogen required as well as plant costs for gas treatment. The shorter process time also has a positive effect on energy requirements. Steelmaking by hydrogen plasma smelting reduction combines the currently predominant route via the blast furnace (BF) and the basic oxygen (BOF). In this process, the iron ore does not have to be agglomerated but can be used directly as fine ore. By eliminating the agglomeration process, a lot of energy will be saved. In addition, the fines fraction, which is a by-product of lump ore processing, can also be used economically. The current analysis helps with process development and creates a basis for comparing different reduction conditions in terms of kinetics in order to develop the process to industrial scale in the next 15 to 20 years.

### 5.2. Outlook

For further experiments on the aspects of this work, a constant and more precise supply system for the fine ore is necessary. To obtain faster and thus more accurate analyses of the exhaust gas flow, the measuring interval of the mass spectrometer and the total volume of the reactor must be reduced. To enable a CO_2_-free steel production in the future, it must be determined if these results are scalable to the bench scale plasma reactor. For this purpose, this test campaign must be adapted for the HPSR plant at the voestalpine Stahl Donawitz GmbH.

## Figures and Tables

**Table 1:** Preliminary testing program.

Experiment Designation	Speed of the Charging System (-)	Total Gas Flow (Nl/min)	Hydrogen Content (%)
Ex. 1–1	15	5	40
Ex. 1–2	25	5	40
Ex. 1–3	35	5	40
Ex. 1–4	45	5	40
Ex. 1–5	55	5	40
Ex. 1–6	65	5	40
Ex. 2–1	45	5	10
Ex. 2–2	45	5	20
Ex. 2–3	45	5	30
Ex. 2–4	45	5	40

**Table 2:** Main experimental program.

Experiment Designation	Speed of the Charging System (-)	H_2_ Content Pre-/Post-Reduction (%)	H_2_ Content Charging (%)	Aimed Pre-Reduction Degree (%)
Ex. 3–1	45	40	20	30
Ex. 3–2	65	40	40	30
Ex. 3–3	45	40	40	55
Ex. 3–4	15	40	40	90
Ex. 3–5	45	40	10	20
Ex. 3–6	45	40	30	35
Ex. 3–7	25	40	20	50

**Table 3:** Chemical composition of the Carajas iron ore.

No	Element	(wt.%)
1	Fe_2_O_3_ ^1^	92.83
2	FeO	1.07
3	Total Fe	65.81
4	Silica	1.694
5	Aluminum oxide	1.01
6	Manganese	0.17
7	Phosphorus	0.057
8	Total sulfur	0.014
9	LOI ^2^	2.79

^1^ Calculated value after analysis; ^2^ Loss of ignition.

**Table 4:** The grain size distribution of Carajas iron ore.

Mesh Size (μm)	Fraction (wt.%)	Cum (wt.%)
63–125	50	50
25–63	50	100

**Table 5:** Chemical composition of ignition pin and steel crucible.

Element	Unit	C	Si	Mn	P	S	Cr	Mo	Ni	Al	Cu
Steel crucible	(wt.%)	0.178	0.261	1.325	0.009	0.005	0.083	0.031	0.168	0.027	0.179
Ignition pin	(wt.%)	0.441	0.217	0.85	0.008	0.028	0.985	0.162	0.085	0.021	0.116

**Table 6:** Purity and residuals of the process gases.

Product Name	Purity (%)	O_2_ (ppm)	H_2_O (ppm)	N_2_ (ppm)
Hydrogen 5.0	≥99.999	≤2	≤5	≤3
Argon 5.0	≥99.999	≤2	≤3	≤5
Nitrogen 5.0	≥99.999	≤3	≤5	-

**Table 7:** Worksheet for the statistical analysis in MODDE^®^ 13 Pro.

	Factors				Responses			
Exp Name	Oxygen Charging Rate (g/min)	H_2_ Content Charging (%)	H_2_ Content Pre-/Post-Reduction (%)	Pre-Reduction Degree (%)	Time for Reduction (RD 85%) (s)	Average η H_2_ Charging (%)	Oxygen Reduction Charging (g/min)	Oxygen Reduction Post-Reduction (g/min)
Ex. 1–1	0.765	40	40	81.2	1245.4	41	0.714	0.602
Ex. 1–2	1.111	40	40	78.9	2318.6	42.5	0.713	0.614
Ex. 1–3	1.397	40	40	71.2	2524.8	50.1	0.784	0.701
Ex. 1–4	1.566	40	40	64.8	2083.8	51.4	0.828	0.815
Ex. 1–5	2.691	40	40	75.9	2672.3	53.7	0.87	0.768
Ex. 1–6	2.816	40	40	73.4	2636.3	50.9	0.854	0.905
Ex. 2–1	1.596	10	10	23.2	5871.5	69.3	0.311	0.396
Ex. 2–2	1.708	20	20	39.2	5317.5	60.4	0.527	0.487
Ex. 2–3	1.868	30	30	54.6	2758.2	57.1	0.68	0.713
Ex. 2–4	1.445	40	40	85.1	2276.7	46	0.794	0.650
Ex. 3–1	1.655	20	40	29.1	2276.9	63.79	0.532	1.129
Ex. 3–2	2.239	40	40	34.5	2161.1	59.3	0.918	0.902
Ex. 3–3	1.8	40	40	58.7	2208.4	49.6	0.807	0.767
Ex. 3–4	0.636	40	40	78.4	2875.4	29.5	0.521	0.494
Ex. 3–5	1.925	10	40	23.5	2028.8	86.3	0.413	1.303
Ex. 3–6	1.853	30	40	37.6	2438.3	63.2	0.734	0.853
Ex. 3–7	1.292	20	40	56	2397.4	63.2	0.487	0.914
Ex. 3–8	3.724	10	40	37.3	1413.1	93.8	0.701	1.424

**Table 8:** Acronyms of the statistical analysis.

Acronym	Meaning
N	Number of valid experiments responses
R2	Coefficient of determination
RSD	Residual standard deviation
DF	Number of degrees of freedom
Q2	Percent of the variation of the response predicted by the model
Interval	Confidence interval

**Table 9:** Experiment evaluation during iron ore feeding.

Parameter	Ex. 1–4	Ex. 1–6	Ex. 3–3
Focal Spot Area A1 (mm^2^)	53.1	39.4	22.9
Focal Spot Area A2 (mm^2^)	30.3	19.3	25.1
Focal Spot Area A3 (mm^2^)	43.9	35.5	74.1
Focal Spot Area A4 (mm^2^)	59.6	52.3	23.5
Focal Spot Area A5 (mm^2^)	56.9	68.8	23.1
Average Spot Area (mm^2^)	48.8	43.0	33.7
Oxygen removal rate (g/min)	0.828	0.854	0.807
Oxygen supply rate (g/min)	1.566	2.816	1.800
Reduction degree (%)	52.8	30.3	44.8
Hydrogen supply rate (L/min)	2	2	2
Specific hydrogen supply (l hydrogen/g oxygen bond to iron)	1.28	0.71	1.11
Partial pressure p_H2_ (kPa)	40.52	40.52	40.52
Partial pressure p_H2O_ (kPa)	10.98	10.92	10.75
GOD/Hydrogen Utilization (-)	0.514	0.509	0.496
Equilibrium partial pressure ratio K’_H_ (-)	1.057	1.038	0.986
Reaction rate r (kg O_2_/m^2^ s)	0.28	0.33	0.40
Reaction rate constant k_a_ (kg O_2_/m^2^ s Pa)	9.39 × 10^−6^	1.10 × 10^−5^	1.35 × 10^−5^
O/Fe (-)	0.71	1.05	0.83
Estimated temperature (°C)	3035	3025	3015

## Data Availability

The data presented in this study are available within the article.
